# Functional Granulocyte–Macrophage Colony-Stimulating Factor (GM-CSF) Delivered by Canine Histiocytic Sarcoma Cells Persistently Infected with Engineered Attenuated Canine Distemper Virus

**DOI:** 10.3390/pathogens12070877

**Published:** 2023-06-27

**Authors:** Katarzyna Marek, Federico Armando, Thanaporn Asawapattanakul, Vanessa Maria Nippold, Philippe Plattet, Gisa Gerold, Wolfgang Baumgärtner, Christina Puff

**Affiliations:** 1 Department of Pathology, University of Veterinary Medicine Hannover, Foundation, 30559 Hannover, Germany; 2 Center for Systems Neuroscience, University of Veterinary Medicine Hannover, Foundation, 30559 Hannover, Germany; 3 Division of Experimental Clinical Research, Vetsuisse University Bern, 3012 Bern, Switzerland; 4 Department of Biochemistry, University of Veterinary Medicine Hannover, Foundation, 30559 Hannover, Germany; 5 Research Center for Emerging Infections and Zoonoses (RIZ), University of Veterinary Medicine Hannover, Foundation, 30559 Hannover, Germany; 6 Wallenberg Centre for Molecular Medicine (WCMM), Umeå University, 901 87 Umeå, Sweden; 7 Department of Clinical Microbiology, Virology, Umeå University, 901 87 Umeå, Sweden

**Keywords:** canine distemper virus, DH82 cells, genetically engineered viruses, GM-CSF, histiocytic sarcoma, viral oncolysis

## Abstract

The immune response plays a key role in the treatment of malignant tumors. One important molecule promoting humoral and cellular immunity is granulocyte–macrophage colony-stimulating factor (GM-CSF). Numerous successful trials have led to the approval of this immune-stimulating molecule for cancer therapy. However, besides immune stimulation, GM-CSF may also accelerate tumor cell proliferation, rendering this molecule a double-edged sword in cancer treatment. Therefore, detailed knowledge about the in vitro function of GM-CSF produced by infected tumor cells is urgently needed prior to investigations in an in vivo model. The aim of the present study was to functionally characterize a persistent infection of canine histiocytic sarcoma cells (DH82 cells) with the canine distemper virus strain Onderstepoort genetically engineered to express canine GM-CSF (CDV-Ond^neon-GM-CSF^). The investigations aimed (1) to prove the overall functionality of the virally induced production of GM-CSF and (2) to determine the effect of GM-CSF on the proliferation and motility of canine HS cells. Infected cells consistently produced high amounts of active, pH-stable GM-CSF, as demonstrated by increased proliferation of HeLa cells. By contrast, DH82 cells lacked increased proliferation and motility. The significantly increased secretion of GM-CSF by persistently CDV-Ond^neon-GM-CSF^-infected DH82 cells, the pH stability of this protein, and the lack of detrimental effects on DH82 cells renders this virus strain an interesting candidate for future studies aiming to enhance the oncolytic properties of CDV for the treatment of canine histiocytic sarcomas.

## 1. Introduction

Histiocytic sarcoma (HS), which can occur in a disseminated or localized form, is a malignant tumor in humans and dogs that has a comparable poor prognosis in both species. Due to the higher prevalence of canine HS compared with its human counterpart, dogs are an interesting translational model for this neoplastic disease [1,2,3,4,5].

Treatment options for canine HS are still very limited [1,2,3,4,5]. This malignant neoplasm is characterized by short survival times, with a median of 39 days after diagnosis in palliatively treated dogs [1]. Therefore, there is an urgent need to find new treatment options, and applying immunotherapy is one possible option [6]. 

Granulocyte–macrophage colony-stimulating factor (GM-CSF) is a link between the innate and adaptive immune systems. It is a highly effective cytokine and one of the most commonly inserted genes in oncolytic viruses [7]. GM-CSF has already been inserted into adenovirus, herpes simplex virus, new castle disease virus, vaccinia virus, measles virus (MV), and, more recently, canine distemper virus [8,9,10,11,12]. GM-CSF is the first cancer immunotherapeutic approved by the U.S. Food and Drug Administration (FDA) [13]. Moreover, Talimogene laherparepvec (TVEC), a genetically modified herpes simplex virus expressing GM-CSF, was the first FDA-approved virus for oncolytic therapy. The application of GM-CSF in tumors can take place in different forms, such as naked DNA, peptide, protein, antigen-loaded dendritic cells, and whole cells [14]. GM-CSF can be administered systemically or locally, either directly or as an anti-cancer vaccine or GM-CSF-producing oncolytic virus [15,16,17,18]. The mode of action of GM-CSF is complex. Anti-tumoral effects result from direct inhibitory effects on tumor cells, the modulation of the immune response, and the inhibition of angiogenesis [13,19,20]. Interestingly, GM-CSF induces cell arrest at the G0/G1 phase and thus has a direct inhibitory effect on cell proliferation, as demonstrated for colorectal, breast, and non-small cell lung cancer [16]. Moreover, it has been observed that GM-CSF promotes the maturation and differentiation of numerous immune cells, such as granulocytes (neutrophils and eosinophils), monocytes, and antigen-presenting cells, and it acts indirectly on CD4^+^ and CD8^+^ T lymphocytes [11,13,16,21,22,23].

The presence of GM-CSF leads to the polarization of tumor-associated macrophages (TAMs) into pro-inflammatory M1 macrophages, which release anti-tumoral cytokines [16,24]. In a mouse model, measles virus (MV) with the insertion of GM-CSF enhanced tumor volume reduction in human lymphoma xenografts compared with non-modified MV [22]. Interestingly, this effect was attributed to increased intratumoral infiltration by neutrophils [22]. A similar increase in intratumoral inflammatory cell infiltrates, mainly CD4^+^ and CD8^+^ T cells, was observed in a syngeneic colon adenocarcinoma model in immunocompetent mice [11]. The latter study also showed long-term protection against tumor re-engraftment [11]. Although numerous studies have emphasized the strong anti-tumoral effects of GM-CSF treatment [8,11,22,25,26,27], other studies have described detrimental effects on tumor cell growth [16,21,28]. 

Canine distemper virus (strain Onderstepoort; CDV-Ond), a morbillivirus closely related to MV, has already demonstrated promising anti-tumor effects against canine histiocytic sarcoma in vitro and in a murine xenotransplantation model [10,29,30,31,32,33,34,35]. In a murine xenotransplantation model using canine histiocytic sarcoma cells (DH82 cells), persistently CDV-infected transplants exhibited complete spontaneous regression [33], while acutely infected neoplasms showed only transient growth retardation [35]. In acutely infected xenotransplants, the increased infiltration of macrophages was noted, which might represent an anti-tumoral immune response [35]. To enhance this effect, a CDV-Ond strain was genetically engineered to express canine GM-CSF (CDV-Ond^neon-GM-CSF^ [10,36]). CDV-Ond^neon-GM-CSF^ infects DH82 cells at a high rate, and the infected cells produce GM-CSF [10]. 

The aim of the present study was to functionally characterize canine GM-CSF secreted by DH82 cells persistently infected with CDV-Ond^neon-GM-CSF^ in vitro.

## 2. Materials and Methods 

### 2.1. Cell Culture

Non-infected canine histiocytic sarcoma cells (DH82 cells) obtained from the European Collection of Authenticated Cell Cultures (ECACC No. 94062922) were cultured in Minimal Essential Medium (MEM) with Earle’s salts (Merck, Darmstadt, Germany) supplemented with 10% fetal bovine serum (Capricorn Scientific, Ebsdorfergrund, Germany), 1% penicillin/streptomycin (Sigma-Aldrich, Taufkirchen, Germany), and 1% non-essential amino acids (Sigma-Aldrich, Taufkirchen, Germany). DH82 CDV-Ond pi and DH82 cells persistently infected with CDV-Ond^neon^ and CDV-Ond^neon-GM-CSF^ were obtained as previously described [34]. Human cervical carcinoma cells (HeLa cells) were cultured in Dulbecco’s Modified Eagle Medium (DMEM; Merck, Darmstadt, Germany) supplemented with 10% fetal bovine serum (Capricorn Scientific, Ebsdorfergrund, Germany) and 1% penicillin/streptomycin (Sigma-Aldrich, Taufkirchen, Germany). All cells were cultured in T75 flasks (ThermoFischer Scientific, Schwerte, Germany) in an incubator at standard conditions (37 °C, 5% CO_2_, in a water-saturated atmosphere). The medium was changed every 2–3 days.

### 2.2. Virus Neutralization

Supernatants from non-infected DH82 cells and DH82 cells persistently infected with canine distemper virus (CDV) strain Onderstepoort (CDV-Ond), along with modified strains of CDV-Ond with the insertion of mNeonGreen (CDV-Ond^neon^) and CDV-Ond with the insertion of mNeonGreen and canine GM-CSF (CDV-Ond^neon-GM-CSF^), were obtained as described before [10]. Supernatants obtained from DH82 cells persistently infected with CDV-Ond, CDV-Ond^neon^, and CDV-Ond^neon-GM-CSF^ and those from non-infected controls were harvested 7 days after seeding and centrifuged (700× *g*, 10 min, 4 °C). Afterward, the pH of the supernatants was lowered with HCl to a pH of 2 for 30 min to inactivate the CDV. Afterward, NaOH was added to increase the pH of all the supernatants to 7. The measurements of pH were performed using pH indicator strips (Merck, Darmstadt, Germany).

### 2.3. Virus Titration

Virus titration was performed in triplicate to confirm the inactivation of CDV as previously described [37]. Briefly, supernatants of non-infected DH82 cells and DH82 cells persistently infected with CDV-Ond, CDV-Ond^neon^, and CDV-Ond^neon-GM-CSF^ were centrifuged (700× *g*, 10 min, 4 °C). Ten-fold dilutions of the supernatants were prepared in DMEM (Merck, Darmstadt, Germany) containing 10% fetal bovine serum (Capricorn Scientific, Ebsdorfergrund, Germany), 1% penicillin/streptomycin (Sigma-Aldrich, Taufkirchen, Germany), and phleomycin D1 (Zeocin^®^; InvivoGen, Toulouse, France). The titration was performed in 96-well microtiter plates (ThermoFischer Scientific, Schwerte, Germany) containing Vero.DogSLAM cells (1.5 × 10^4^ cells/well). Cells were incubated with the acidified supernatants for 7 days, and the cytopathic effect (CPE) was evaluated daily. The 50% log10 tissue culture infectious dose per milliliter (TCID_50_/mL) of cell-free virus was calculated according to the Reed and Muench method [38].

### 2.4. Immunofluorescence

In order to verify a CDV infection or confirm the successful inactivation, Vero.DogSLAM cells were immunolabelled after virus titration using an antibody directed against CDV nucleoprotein as previously described [10]. Briefly, cells were fixed with 4% buffered paraformaldehyde (PFA 4%, pH 7.4) and permeabilized with PBS-Triton X (0.025%). After serum blocking, cells were incubated overnight at 4 °C with the anti-CDV nucleoprotein antibody (Table 1). Afterward, cells were washed with PBS/0.1% Triton and incubated for 2 h with the secondary antibody. For nuclear staining, bisbenzimide (Merck, Darmstadt, Germany) was used. Negative controls included the omission of primary or secondary antibodies. Pictures were taken at 200× magnification using a microscope (Olympus IX-70, Olympus Optical Co. GmbH, Hamburg, Germany) equipped with an Olympus DP-72 camera and Olympus cellSens standard software version 2.3 (Olympus optical Co. GmbH, Hamburg, Germany). 

### 2.5. RNA Isolation and cDNA Synthesis

Total RNA was isolated using the RNeasy Mini Kit (Qiagen, Hilden, Germany) with on-column DNA digestion with RNase free DNase (Qiagen, Hilden, Germany) according to the manufacturer’s protocols. The RNA concentration was spectroscopically measured at 260 nm using the GeneQuant pro (GE Healthcare, Amersham, Buckinghamshire, UK). RNA to cDNA transcription was performed according to the manufacturer’s protocols with OmniScript (Qiagen, Hilden, Germany), RNAseOut (ThermoFischer Scientific, Schwerte, Germany), and Random Hexamers (Promega, Madison, WI, USA). Reverse transcription was performed using a Biometra Thermocycler T-Gradient ThermoBlock (American Laboratory Trading, East Lyme, CT, USA) under the following conditions: 25 °C for 10 min, 37 °C for 1 h, and 93 °C for 5 min.

### 2.6. Primer Design

The primers for qualitative and quantitative RT-PCR for the detection of CDV nucleoprotein mRNA transcripts were taken from the literature [34]. The primers used for qualitative CDV nucleoprotein RT-PCR and quantitative CDV RT-PCR are presented in Table 2.

### 2.7. Reverse Transcription Quantitative PCR (RT-qPCR)

The NucleoSpin^®^ Gel and PCR Clean-up Kit (Macherey-Nagel, Düren, Germany) was used for the isolation of PCR amplicons from the agarose gel according to the manufacturer’s protocol. Standard curves for the estimation of copy numbers were generated using RT-PCR amplicons in a serial dilution from 10^8^ to 10^2^ copies/µL. RT-qPCR was performed with four samples per condition and negative controls, as previously described [34]. Quantitative PCR was performed using the Brilliant III Ultra-Fast SYBR® Green QPCR Master Mix (Agilent Technologies, Cedar Creek, TX, USA). Primers were diluted to a final concentration of 150 nM each. The detection of CDV mRNA transcripts was performed using the AriaMx Real-time PCR System (Agilent Technologies, Santa Clara, CA, USA) at the following conditions: denaturation at 95 °C for 3 min, 35 cycles at 95 °C for 5 s, and 57 °C for 10 s, followed by a melting curve consisting of one initial denaturation step at 95 °C for 30 s followed by 65 °C, increasing the temperature by 1 °C per cycle. 

### 2.8. Immunoblotting

The immunoblotting of supernatants obtained from non-infected DH82 cells or DH82 cells infected with CDV-Ond, CDV-Ond^neon^, and CDV-Ond^neon-GM-CSF^ was carried out in three independent samples, as previously described [10,29]. The standardization of protein concentration was performed using the Pierce^TM^ BCA Protein Assay Kit (Thermo Scientific, Schwerte, Germany). The same amount of protein was separated on 15% (immunoblotting of GM-CSF) or 6% (immunoblotting of CDV) SDS-PAGE gels. The proteins were transferred to a nitrocellulose membrane (Bio-Rad, Hercules, CA, USA) and blocked with 5% milk blocking buffer (Merck, Darmstadt, Germany) in 0.1% Tween 20 in phosphate-buffered saline (PBS) for 1 h. Immunoblotting was performed using a primary antibody directed against canine GM-CSF (goat, polyclonal, 1:1000; R&D Systems, Minneapolis, MN, USA), CD116 (rabbit, polyclonal, 1:500; Invitrogen, CA, USA), and CDV nucleoprotein (NP) (mouse, monoclonal, clone D110, 1:1000; University of Bern, Prof. Zurbriggen), respectively. Horseradish peroxidase (HRP)-conjugated rabbit anti-goat (1:1000; R&D Systems; Minneapolis, MN, USA), goat anti-rabbit (1:1000; Thermo Scientific, Schwerte, Germany), and rabbit anti-mouse (1:1000; Invitrogen, CA, USA) antibodies were used as secondary antibodies for the detection of GM-CSF, GM-CSFR, and CDV nucleoprotein, respectively. Protein bands were visualized using a chemiluminescent substrate (SuperSignalTM West Pico PLUS, Thermo Scientific, Schwerte, Germany) and a ChemiDoc MP Imaging System (Bio-Rad, Hercules, CA, USA). Densitometric analysis was performed to quantify the band sizes and intensities using ImageJ version 1.51.0 (https://imagej.nih.gov/ij/). 

### 2.9. Immunohistochemistry

Immunohistochemistry for the GM-CSF receptor (CD116) was performed to investigate the expression in DH82 cells. Therefore, formalin-fixed, paraffin-embedded cell pellets obtained from non-infected DH82 cells and DH82 cells persistently infected with CDV-Ond, CDV-Ond^neon^, and CDV-Ond^neon-GM-CSF^ were used. HeLa cell pellets with a known expression of the GM-CSF receptor (CD116) served as positive controls. Immunohistochemistry was carried out as previously described [10]. Immunolabeling was performed in triplicate with negative controls, as previously described [10]. Briefly, after the dewaxing, rehydration, and blocking of endogenous peroxidases, sections were blocked with goat serum for 30 min. Subsequently, slides were incubated overnight at 4 °C with the primary GM-CSF receptor (CD116) antibody (rabbit, polyclonal, 1:500; Invitrogen, CA, USA). Afterward, a secondary goat anti-rabbit biotinylated antibody (1:200, Vector Laboratories, Burlingame, CA, USA) and an avidin–biotin complex (ABC) peroxidase kit (Vectastain® Elite® ABC Kit, Vector Laboratories, Burlingame, CA, USA) were applied for 30 min and 20 min, respectively. A 3′3′-diaminobenzidine (DAB) system (Vector Laboratories, Burlingame, CA, USA) was used for the detection of positive reactions (Table 1). Nuclei were counterstained with Mayer’s hemalum (Carl Roth GmbH, Karlsruhe, Germany). For the negative controls, the specific primary antibody was replaced by normal rabbit serum. The dilution of the negative controls was chosen according to the protein concentration of the replaced primary antibodies. HeLa cell pellets were used as a positive control. Pictures were taken at 400× magnification using a microscope (Olympus BX51, Olympus Optical Co. GmbH, Hamburg, Germany) equipped with an Olympus D72 camera (Olympus Optical Co. GmbH, Hamburg, Germany). CD116 immunolabeling was qualitatively analyzed.

### 2.10. Scratch Wound Assay

The scratch assay was performed as previously described [29]. Briefly, non-infected DH82 cells were seeded at a density of 0.3 × 10^5^ cells/well into 96-well microtiter plates (ThermoFischer Scientific, Schwerte, Germany) with 200 mL of Minimal Essential Medium (MEM) with Earle’s salts (Merck, Darmstadt, Germany) supplemented with 10% fetal bovine serum (Capricorn Scientific, Ebsdorfergrund, Germany), 1% penicillin/streptomycin (Sigma-Aldrich, Taufkirchen, Germany) and 1% non-essential amino acids (Sigma-Aldrich, Taufkirchen, Germany). Cells were cultured for 2 days under standard conditions (37 °C, 5% CO_2_, water-saturated atmosphere). When cells reached 99% confluence, the cell monolayer was scratched in a straight line with a p100 pipette tip. The medium was discarded, and the cell monolayer was washed with a washing medium (Minimal Essential Medium (MEM) with Earle’s salts (Merck, Darmstadt, Germany)). Acidified supernatants obtained from non-infected DH82 cells or DH82 cells infected with CDV-Ond, CDV-Ond^neon^, and CDV-Ond^neon-GM-CSF^, respectively, were added to the scratched wells. Culture supernatant supplemented with 2 ng/mL of canine GM-CSF (R&D Systems, Systems, Minneapolis, MN, USA) was used for additional samples. Pictures were taken at the same position directly after performing the scratch (T_0_) and after 24 h (T_24_) using a phase contrast microscope (Olympus IX-70, Olympus Optical Co. GmbH, Hamburg, Germany) equipped with an Olympus DP-72 camera and Olympus cellSens standard software version 2.3 (Olympus Optical Co. GmbH, Hamburg, Germany). The percentage of cell-free area was calculated with ImageJ 1.52p according to the following formula: 100 − percentage of cell-covered area, as previously described [39]. The change in the cell-covered area was calculated according to the following formula: (|Area T_0_ − Area T_24_|)/Area T_0_ ∗ 100. 

### 2.11. Cell Duplication Assay

DH82 cells were seeded at a density of 2 × 10^4^ cells in quadruplicate in 24-well plates in MEM medium (Merck, Darmstadt, Germany) with the addition of recombinant human GM-CSF (R&D Systems, Minneapolis, MN, USA), canine GM-CSF (R&D Systems, Minneapolis, MN, USA), or acidified supernatants obtained from non-infected DH82 cells and DH82 cells persistently infected with CDV-Ond, CDV-Ond^neon^, and CDV-Ond^neon-GM-CSF^, respectively. Commercially available human and canine GM-CSF was supplemented at 5 µg/mL. A culture medium supplemented with caGM-CSF or rhGM-CSF was used in an amount of 500 µL/well. The acidified supernatants obtained from DH82 cells infected with CDV-Ond, CDV-Ond^neon^, or CDV-Ond^neon-GM-CSF^ were diluted to 2:3 with MEM. Cell numbers were quantified after 6 and 12 h. Therefore, cells were detached from the bottom of the well with 0.05% trypsin/0.02% EDTA (Sigma-Aldrich, Taufkirchen, Germany). Trypsinized cell suspensions were centrifuged for 10 min at 250× *g* at 4 °C. The obtained cell pellets were re-suspended in 30 µL MEM medium, and 20 µL of the cell suspension was added to 40 µL of trypan blue to obtain a final 1:2 dilution. Counting was performed using a 0.100 mm Neubauer counting chamber (Assistent, Sondheim vor der Rhön, Germany) and a light microscope (Axiovert 10, Zeiss, Oberkochen, Germany) [40]. The cell number was obtained according to the following formula:

(number of counted cells × dilution factor)/number of counted squares × volume of one square mm^3^ = number of cells/mL (number of counted cells × 0.33)/3 × 0.1 mm^3^ = number of cells/mL

HeLa cells were seeded at a density of 2 × 10^4^ cells in quadruplicate in 24-well plates in DMEM (Merck, Darmstadt, Germany) with the addition of recombinant human GM-CSF (R&D Systems, Minneapolis, MN, USA), canine GM-CSF (R&D Systems, Minneapolis, MN, USA), or acidified supernatants obtained from non-infected DH82 cells and DH82 cells persistently infected with CDV-Ond, CDV-Ond^neon^, and CDV-Ond^neon-GM-CSF^, respectively. Commercially available human and canine GM-CSF was supplemented at 5 µg/mL. A culture medium supplemented with caGMCSF or rhGM-CSF was used in an amount of 500 µL/well. The acidified supernatants obtained from DH82 cells infected with CDV-Ond, CDV-Ond^neon^, or CDV-Ond^neon-GM-CSF^ were diluted to 2:3 with DMEM. The cell number was quantified after 6 and 12 h. Thereafter, cells were detached from the bottom of the well with 0.05% trypsin/0.02% EDTA (Sigma-Aldrich, Taufkirchen, Germany). Trypsinized cell suspensions were centrifuged for 5 min at 300× *g* at 4 °C. Obtained cell pellets were re-suspended in 30 µL of DMEM, and 20 µL of the cell suspension was added to 40 µL of trypan blue to obtain a final 1:2 dilution. Counting was performed as described above.

### 2.12. Statistical Analysis

For descriptive statistics, the median and range were calculated. For the analysis of data obtained from the virus titration, immunofluorescence, RT-qPCR, and immunoblotting and proliferation assays, the non-parametric Mann–Whitney U and Wilcoxon signed-rank tests were used. Statistical analysis was performed with SAS software version 7.1.5.0 (SAS Institute, Cary, NC, USA, www.sas.com). The level of significance was set at *p* ≤ 0.05. Graph creation was carried out using GraphPadPrism version 8.0.1 for Windows (GraphPad Software, La Jolla, CA, USA, www.graphpad.com).

## 3. Results

### 3.1. Acidic Inactivation of the Supernatant Results in CDV Neutralization

**Figure 1:**
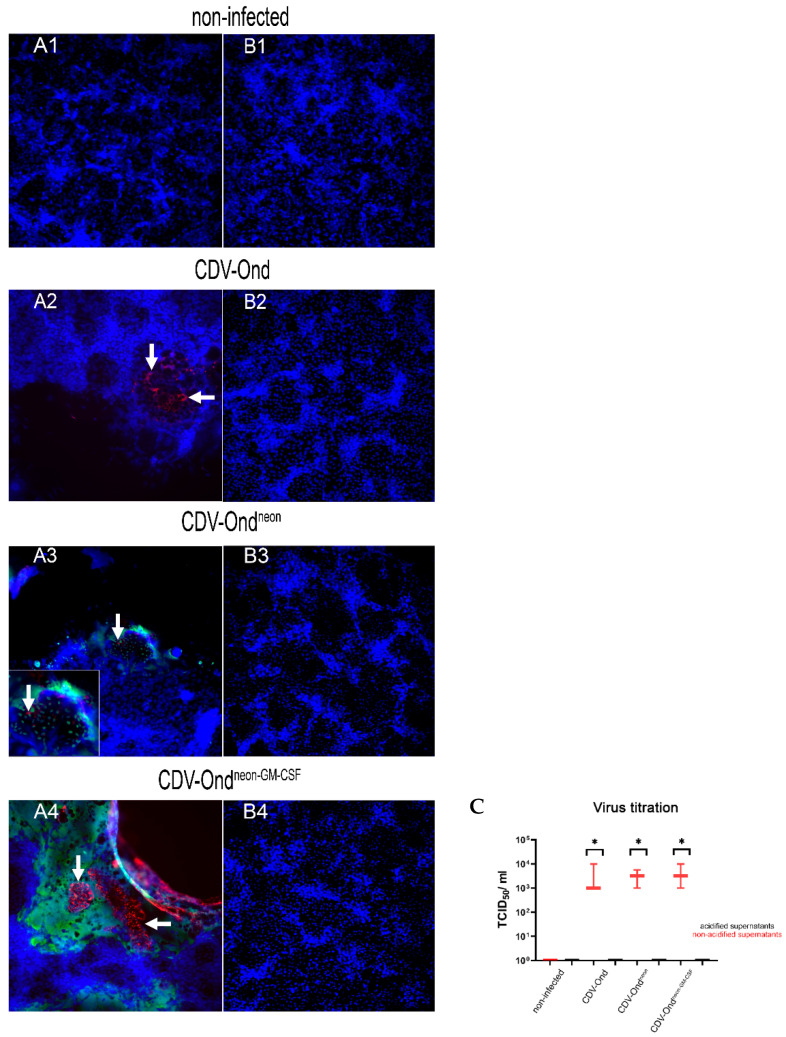
Virus titration on Vero.DogSLAM cells. Immunofluorescence for CDV nucleoprotein before and after acidification of supernatants obtained from non-infected DH82 cells and DH82 cells persistently infected with CDV-Ond, CDV-Ond^neon^, and CDV-Ond^neon-GM-CSF^. Titration with supernatants without (**A1**) or after acidification (**B1**) of non-infected controls revealed no CDV nucleoprotein-positive Vero.DogSLAM cells. Virus titration with supernatants of DH82 cells persistently infected with CDV-Ond (**A2**), CDV-Ond^neon^ (**A3**), and CDV-Ond^neon-GM-CSF^ (**A4**) displayed CDV nucleoprotein-positive cells (red). After acidification, supernatants of CDV-Ond (**B2**), CDV-Ond^neon^ (**B3**), and CDV-Ond^neon-GM-CSF^ (**B4**) revealed no CDV nucleoprotein-positive cells. Nuclei were counterstained with bisbenzimide (blue). Additionally, Vero.DogSLAM cells infected with native supernatants of CDV-Ond^neon^ (**A3**) and CDV-Ond^neon-GM-CSF^ (**A4**) revealed mNeonGreen-positive cells (green). Graphical presentation of the TCID_50_/mL in native and acidified supernatants of all analyzed supernatants, as determined by virus titration (**C**). Supernatants obtained from CDV-infected cultures showed significantly lower TCID_50_/mL after acidification, independent of the virus strain. White arrows indicate positive cells. Box plots represent minimum, median, and maximum. Significant differences between the treatment states (Wilcoxon signed-rank test) are labeled by asterisks: * = *p* ≤ 0.05; n = 3 per condition and group.

Virus replication was comparatively investigated using virus titration, which revealed no significant differences in the titers produced by DH82 cells infected with CDV-Ond, CDV-Ond^neon^, and CDV-Ond^neon-GM-CSF^ (Mann–Whitney U test; *p* > 0.05; Figure 1). After inactivation by acidification (pH 2), a significant reduction in the 50% log10 tissue culture infectious dose per milliliter (TCID_50_/mL) was observed compared with non-acidified supernatants independent of the virus strain (Wilcoxon signed-rank test; *p* ≤ 0.05, Figure 1). In acidified supernatants from CDV-infected cultures, neither a cytopathic effect nor cells immunopositive for CDV nucleoprotein were observed, independent of the virus strain (Table 3, Figure 1). 

**Figure 2:**
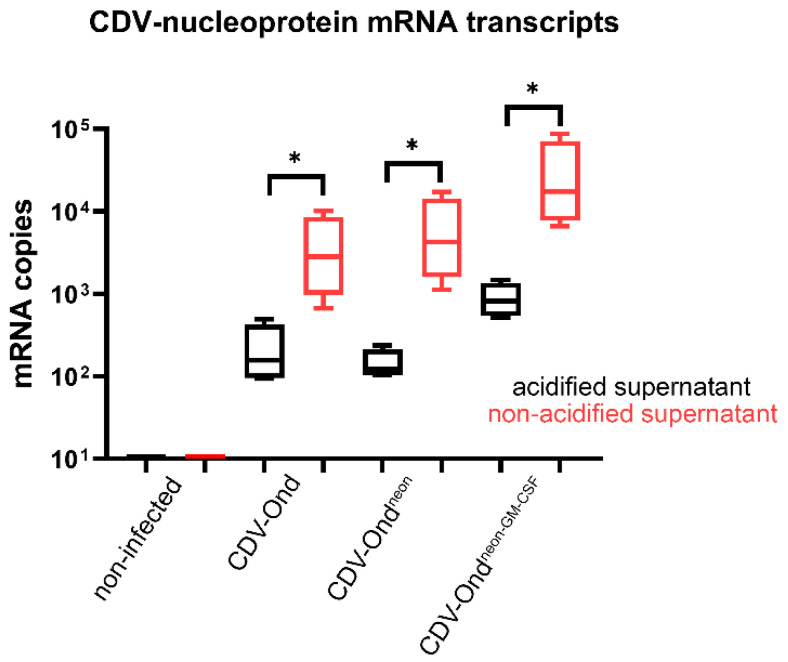
Number of CDV nucleoprotein mRNA transcripts in supernatants obtained from non-infected controls and DH82 cells persistently infected with CDV-Ond, CDV-Ond^neon^, and CDV-Ond^neon-GM-CSF^. The number of CDV mRNA transcripts was significantly decreased in acidified compared with native supernatants. Box plots represent minimum, first quartile, median, third quartile, and maximum. Significant differences (Wilcoxon signed-rank test) are labeled by asterisks, * = *p* ≤ 0.05; n = 4 per condition and group.

At the molecular level, the number of CDV nucleoprotein mRNA transcripts was similar in the supernatants obtained from DH82 cells persistently infected with CDV-Ond, CDV-Ond^neon^, and CDV-Ond^neon-GM-CSF^, respectively (Mann–Whitney U test; *p* > 0.05). After acidification, the number of CDV nucleoprotein mRNA transcripts was significantly lower in supernatants from all treated cultures compared with corresponding native controls (CDV-Ond: *p* = 0.0152, CDV-Ond^neon^: *p* = 0.0259 and CDV-Ond^neon-GM-CSF^, *p* = 0.0152; Wilcoxon signed-rank test; Figure 2 and Table 4). In supernatants of non-infected DH82 cells, CDV nucleoprotein mRNA transcripts were not present.

### 3.2. Acidic Inactivation of the Supernatant Does Not Affect the GM-CSF Protein Content 

**Figure 3:**
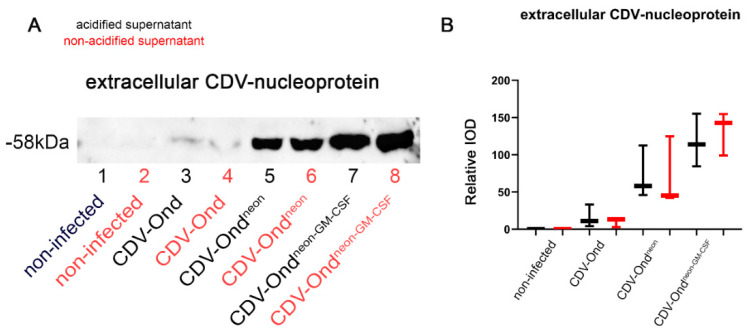
Western blot analysis of CDV nucleoprotein in supernatants. (**A**) Immunoblotting with an anti-CDV nucleoprotein antibody revealed bands at 58 kDa in native and acidified supernatants obtained from DH82 cells infected with CDV-Ond, CDV-Ond^neon^, and CDV-Ond^neon-GM-CSF^, while supernatants from non-infected controls were negative. (**B**) Densitometric analysis of the amount of CDV nucleoprotein in supernatants obtained from non-infected DH82 cells or DH82 cells infected with CDV-Ond, CDV-Ond^neon^, and CDV-Ond^neon-GM-CSF^. Box plots represent minimum, median, and maximum. IOD—integrated optical density; n = 3 per condition and group.

Immunoblotting with an anti-CDV nucleoprotein antibody displayed protein bands at 58 kDa in supernatants from DH82 cells infected with CDV-Ond, CDV-Ond^neon^, and CDV-Ond^neon-GM-CSF^, while no bands were present in non-infected supernatants. Supernatants obtained from DH82 cells infected with CDV-Ond contained significantly lower amounts of CDV nucleoprotein than supernatants obtained from CDV-Ond^neon^-infected cultures (*p* = 0.0404) and CDV-Ond^neon-GM-CSF^-infected cultures (*p* = 0.0404) (Mann–Whitney U test; Table 3, Figure 3). Similar results were obtained for acidified supernatants with significantly lower amounts of CDV nucleoprotein in supernatants obtained from CDV-Ond-infected DH82 cells compared with supernatants obtained from cultures infected with both other virus strains (CDV-Ond^neon^: *p* = 0.0404; CDV-Ond^neon-GM-CSF^: *p* = 0.0404; Mann–Whitney U test; Table 5, Figure 3). The comparison of native compared with corresponding acidified supernatants showed no significant differences in the amount of CDV nucleoprotein (Wilcoxon signed-rank test; *p* > 0.05). 

**Figure 4:**
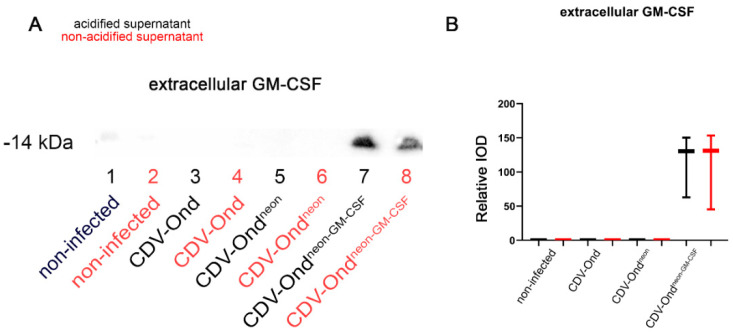
Western blot analysis of GM-CSF in supernatants using an anti-GM-CSF antibody. (**A**) Immunoblotting with an anti-GM-CSF antibody showed bands at 14 kDa in native and acidified supernatants obtained from DH82 cells infected with CDV-Ond^neon-GM-CSF^. (**B**) Native and acidified supernatants obtained from DH82 cells infected with CDV-Ond^neon-GM-CSF^ contained more GM-CSF than supernatants from all other groups, and there were no significant differences between native and acidified supernatants. n = 3 per condition and group; IOD—integrated optical density.

Immunoblotting with an anti-GM-CSF antibody revealed protein bands at 14 kDa in native and acidified supernatants obtained from DH82 cell cultures infected with CDV-Ond^neon-GM-CSF^. The amount of GM-CSF did not differ between native and acidified supernatants obtained from DH82 cells infected with CDV-Ond^neon-GM-CSF^ (Wilcoxon signed-rank test; *p* > 0.05; Table 6, Figure 4). In supernatants obtained from DH82 cells infected with CDV-Ond, CDV-Ond^neon^, and non-infected controls, GM-CSF was not detectable in native or in acidified samples (Table 4, Figure 4). In native and acidified supernatants of CDV-Ond^neon-GM-CSF^-infected DH82 cells, the amount of GM-CSF was significantly higher than in supernatants from non-infected controls and DH82 cells infected with CDV-Ond and CDV-Ond^neon^ (Mann–Whitney U test; *p* ≤ 0.05).

### 3.3. GM-CSF Produced by CDV-Ond^neon-GM-CSF^-Infected DH82 Cells Is Functional and Does Not Affect DH82 Cell Behavior

After the verification of the presence of GM-CSF in supernatants obtained from DH82 cells persistently infected with CDV-Ond^neon-GM-CSF^ and the confirmation that virus neutralization by acidification did not affect the amount of GM-CSF, the next step was to analyze the effect on DH82 cells and to assess its functionality.

**Figure 5:**
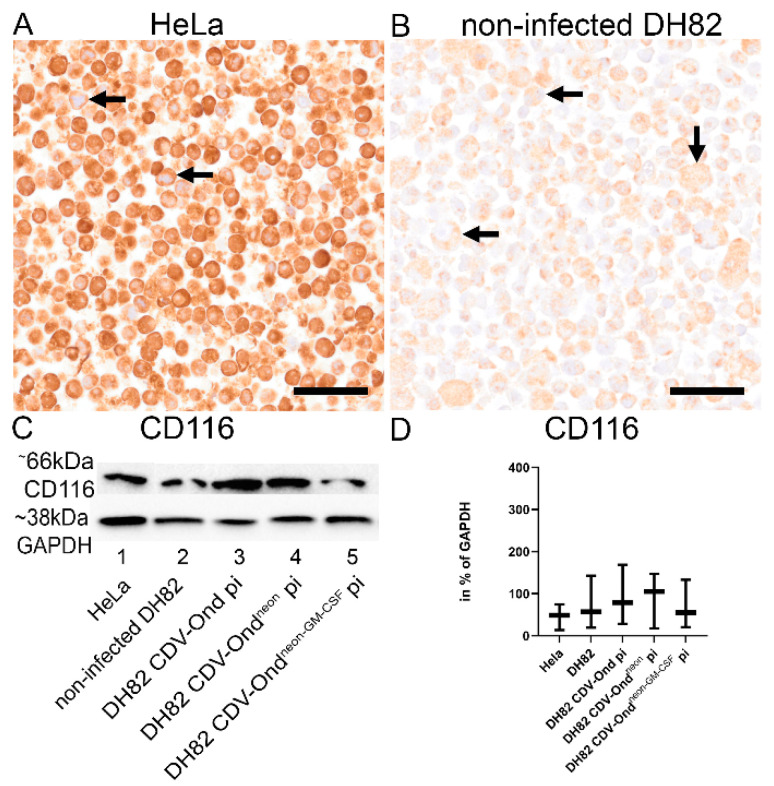
Expression of the GM-CSF receptor (CD116) in HeLa cells, non-infected DH82 cells, and DH82 cells persistently infected with CDV-Ond, CDV-Ond^neon^, and CDV-Ond^neon-GM-CSF^. HeLa cells were used as a positive control and expressed the GM-CSF receptor (CD116) on the cell membrane (**A**). Representative picture of non-infected DH82 cells expressing CD116 on the cell membrane (**B**). Black arrows indicate positive cells. Bars = 400 µm. Western blot analysis of CD116 using an anti-GM-CSF receptor antibody (**C**). Intracellular amount of CD116 in % of GAPDH. Immunoblotting of CD116 showed no significant differences in the sizes and intensities of bands at 66 kDa in HeLa cells, non-infected DH82 cells, and DH82 cells persistently infected with CDV-Ond, CDV-Ond^neon^, and CDV-Ond^neon-GM-CSF^ (**C**). Similarly, the relative protein expression of CD116 did not differ between the different groups. Box plots represent minimum, median, and maximum (Mann–Whitney U test; *p* > 0.05) (**D**). GAPDH was used as a housekeeping protein. n = 3 per condition and group.

To assess the potential growth-stimulating effect of GM-CSF on histiocytic sarcoma cells (DH82 cells), the presence of the GM-CSF receptor (CD116) was investigated in non-infected DH82 cells and DH82 cells persistently infected with CDV-Ond, CDV-Ond^neon^, and CDV-Ond^neon-GM-CSF^. DH82 cells expressed CD116 independently of the infection status, and no significant differences in the CD116 protein amount were observed. (Figure 5; Mann–Whitney U test; *p* > 0.05).

**Figure 6:**
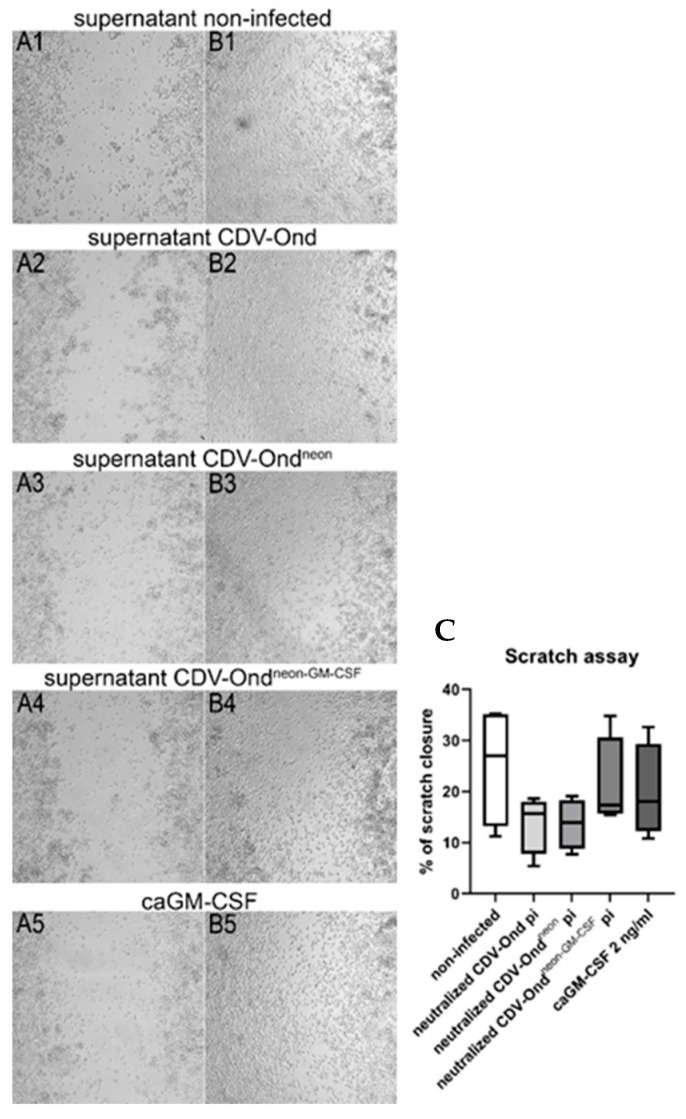
Representative images of the scratch wound assay at time point 0 (**A1**–**A5**) and after 24 h (**B1**–**B5**). The medium of non-infected DH82 cells was supplemented with the acidified medium obtained from non-infected DH82 cells (**A1**) or DH82 cells persistently infected with CDV-Ond (**A2**), CDV-Ond^neon^ (**A3**), CDV-Ond^neon-GM-CSF^(**A4**), or commercially available canine GM-CSF (caGM-CSF; **A5**). The scratch closure was similar in all groups after 24 h of treatment with acidified supernatant obtained from non-infected DH82 cells (**B1**), DH82 cells persistently infected with CDV-Ond (**B2**), CDV-Ond^neon^ (**B3**), CDV-Ond^neon-GM-CSF^ (**B4**), or commercially available canine GM-CSF (caGMCSF; **B5**). Graphical presentation of the percentage of scratch closure (**C**). Box plots represent minimum, first quartile, median, third quartile, and maximum. Mann–Whitney U test revealed no significant differences between the groups: *p* > 0.05. n = 4 per condition and group.

**Figure 7:**
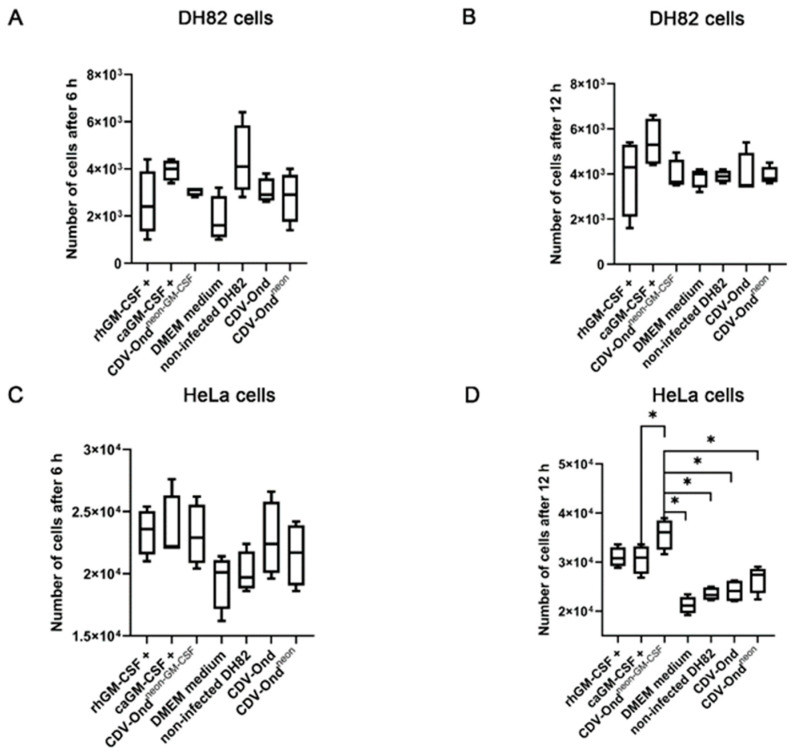
Evaluation of the effect of a GM-CSF supplementation on cell proliferation of DH82 cells and HeLa cells after 6 and 12 h. DH82 cell proliferation was similar in all groups after 6 h (**A**) and 12 h (**B**) independent of the type of supplementation of the medium (*p* > 0.05). (**C**) Cell duplication assay of HeLa cells lacked significant differences between the groups after 6 h. (**D**) After 12 h of substitution with acidified supernatants obtained from CDV-Ond^neon-GM-CSF^-infected DH82 cells, the number of HeLa cells was significantly higher than that in cultures supplemented with commercially available DMEM, acidified supernatants obtained from non-infected DH82 cells, and DH82 cells persistently infected with CDV-Ond or CDV-Ond^neon^ or supplemented with commercially available canine GM-CSF (caGM-CSF). Box plots represent minimum, first quartile, median, third quartile, and maximum. Significant differences (Mann–Whitney U test) are labeled by asterisks * = *p* ≤ 0.05.; n = 4 per time point, condition and group.

To investigate the functional impact of GM-CSF on DH82 cell motility, a scratch wound assay was performed using non-infected DH82 cells and acidified supernatants obtained from non-infected DH82 cells and DH82 cells persistently infected with CDV-Ond, CDV-Ond^neon^, or CDV-Ond^neon-GM-CSF^. Commercially available canine GM-CSF (caGM-CSF) was added to the non-conditioned medium as a control. There were no significant differences in the percentage of scratch closure after 24 h between groups (Mann–Whitney U test; *p* > 0.05; Figure 6). In addition, the effect of GM-CSF on DH82 cell proliferation was examined using a cell duplication assay. The analysis yielded no significant differences among all groups (Mann–Whitney U test; *p* > 0.05, Figure 7). 

For the verification of the overall functionality of GM-CSF produced by CDV-Ond^neon-GM-CSF^-infected DH82 cells, a cell duplication assay was performed with HeLa cells. HeLa cell culture media were supplemented with acidified supernatants obtained from non-infected DH82 cells and DH82 cells persistently infected with CDV-Ond, CDV-Ond^neon^, or CDV-Ond^neon-GM-CSF^. The culture media of HeLa cell control groups were supplemented with commercially available canine and recombinant human GM-CSF (caGM-CSF; rhGM-CSF). After 6 h, the cell duplication assay did not reveal significant differences between the groups (Mann–Whitney U test; *p* > 0.05, Figure 7). Interestingly, after 12 h, the number of cells was significantly higher in HeLa cell samples supplemented with acidified supernatants obtained from DH82 cells persistently infected with CDV-Ond^neon-GM-CSF^ compared with samples supplemented with acidified supernatants obtained from non-infected DH82 cells (*p* = 0.0152) or from DH82 cells infected with CDV-Ond (*p* = 0.0152) or CDV-Ond^neon^ (*p* = 0.0147) or supernatants supplemented with commercial caGM-CSF (*p* = 0.0303). 

Overall, the present study demonstrated that GM-CSF in supernatants obtained from DH82 cells persistently infected with CDV-Ond^neon-GM-CSF^ did not influence histiocytic sarcoma cell proliferation and migration, but at the same time, it exerted its function by stimulating HeLa cell proliferation in vitro.

## 4. Discussion 

The treatment of canine histiocytic sarcomas is often of limited success and leads only to partial or transient regressions or a delay in disease progression [41,42,43]. Therefore, therapeutic advances are greatly needed. Former studies in a murine xenotransplantation model of canine histiocytic sarcomas demonstrated a complete spontaneous regression following the transplantation of persistently CDV-Ond-infected tumor cells. Interestingly, this regression was accompanied by a decreased microvessel density [33], highlighting the importance of agents being able to modulate the tumor microenvironment. One important cytokine known to be involved in angiogenesis and anti-tumoral immune response is GM-CSF [13,16,20]. Therefore, a CDV-Ond strain was genetically engineered to express GM-CSF to enhance the oncolytic properties of this virus [10]. The present study intended to characterize the functionality of GM-CSF produced by CDV-Ond^neon-GM-CSF^-infected canine histiocytic sarcoma cells (DH82 cells) and explore the effect on non-infected canine histiocytic sarcoma cells in vitro. 

To allow an investigation of the effect of GM-CSF produced by CDV-Ond^neon-GM-CSF^-infected DH82 cells, the first step was to inactivate the infectious virus, which was also present in the supernatant of these cultures, to avoid additional CDV infections, which would hamper the interpretation of observed effects. *Morbilliviruses* are stable at pH 4.5–9 [44], whereas GM-CSF maintains its structural stability and thus its functionality in acidic environments [45,46]. Therefore, cell culture supernatants obtained from DH82 cells persistently infected with CDV-Ond^neon-GM-CSF^ were acidified and inactivated at pH 2. Supernatants of all control groups (DH82 cells persistently infected with CDV-Ond and CDV-Ond^neon^, respectively) were treated analogously. Supernatants of non-infected DH82 cell cultures served as an additional control. Successful treatment was confirmed by the absence of cytopathic effects in Vero.DogSLAM cells upon virus titration. Furthermore, a significantly lower number of CDV mRNA transcripts was present in the acidified supernatants of infected cultures, while the protein amount of CDV nucleoprotein remained unchanged. Despite the acidification, the protein amount of GM-CSF did not change significantly compared with untreated supernatants, proving the stability of GM-CSF at low pH values. 

The function of GM-CSF is transmitted by the GM-CSF receptor (CD116), which is composed of subunits α and β, and leads to activation of the Janus Kinase 2 (JAK2)-dependent signaling pathway [16]. The effect of GM-CSF depends on the concentration of available GM-CSF [16,47]. CD116 subunit α binds GM-CSF with a low affinity, whereas subunit β does not bind GM-CSF itself but forms a high-affinity receptor with subunit α [47]. At low concentrations of GM-CSF, phosphatidylinositol 3 kinase (PI3K) and mitogen-activated protein kinases (MAPK) are activated [16]. By contrast, a high concentration of GM-CSF leads to the activation of the signal transducer and activator of the transcription 5 (STAT-5)-dependent pathway [16]. The latter can lead to tumor cell proliferation [16,47,48,49]. Therefore, the next step was to analyze CD116 expression of DH82 cells. All DH82 cells, independently of the infection status, expressed CD116, allowing them to possibly respond to growth stimulation by GM-CSF. This might bear the risk of the unintended stimulation of neoplastic cells as an adverse effect. Interestingly, despite the expression of CD116, the substitution of cell culture media of non-infected DH82 cells with acidified supernatants obtained from CDV-Ond^neon-GM-CSF^-infected DH82 cells or commercially available canine GM-CSF did not result in increased cell proliferation or cell motility. This might be caused by defects in the intracellular signaling pathway since GM-CSF is known to play an important role in myelopoiesis and the function of myeloid cells [16]. However, the lack of a detrimental effect on histiocytic sarcoma cells due to GM-CSF produced by genetically engineered viruses is highly desirable. This might enhance the efficacy of these viruses since GM-CSF can stimulate the non-neoplastic, infiltrating inflammatory cells of the host and evolve an amplified anti-tumor immune response. 

To access the overall functionality of GM-CSF, HeLa cells were used. These cells are known to show increased proliferation after stimulation with GM-CSF [17]. HeLa cells cultured in a medium supplemented with acidified supernatants of DH82 cells persistently infected with CDV-Ond^neon-GM-CSF^ displayed increased proliferation after 12 h of stimulation, confirming the functionality of GM-CSF produced by CDV-Ond^neon-GM-CSF^-infected DH82 cells.

## 5. Conclusions

To summarize, CDV-Ond^neon-GM-CSF^-infected DH82 cells secreted increased amounts of functionally active GM-CSF, while the proliferation and migration of histiocytic sarcoma cells was not influenced. Consequently, this genetic modification might result in an increased were capacity of this CDV-Ond strain compared with that of the non-modified parenteral strain. However, the present results only depict in vitro findings and can only partially mimic the complex interactions in the tumor microenvironment. Therefore, the functionality and efficacy of CDV-Ond^neon-GM-CSF^ need to be further detailed in more complex situations, such as three-dimensional co-cultures of neoplastic cells with other components of the tumor microenvironment, and especially in controlled in vivo situations, such as a murine xenotransplantation model.

## Figures and Tables

**Table 1:** Summary of antibodies used for immunostaining including primary antibodies, host species, clonality, blocking serum, dilution, and secondary antibodies.

Primary Antibody	Host Species, Clonality	Blocking Serum	Dilution	Secondary Antibody (1:200)
CDV-NP (University of Bern, Prof. Zurbriggen)	Mouse, monoclonal, clone D110	Goat serum	1:100 (IF)	GaM-Cy3
CD116 (Invitrogen, CA, USA)	Rabbit, polyclonal	Goat serum	1:500 (IHC)	GaR-b

CDV-NP—canine distemper virus nucleoprotein; GaM-Cy3—goat anti-mouse cyanine 3-conjugated; GaR-b—goat anti-rabbit biotinylated; IF—immunofluorescence; IHC—immunohistochemistry.

**Table 2:** Primers used for qualitative and quantitative RT-PCR, including expected amplicon length, position, and GenBank accession number.

Gene	Primer Sequence (5′-3′)		Amplicon Length (bp)	Position	GenBank Accession Number
CDV ^#^	Forward *	ACAGGATTGCTGAGGACCTAT	287	769–789	AF378705
	Reverse *	CAAGATAACCATGTACGGTGC		1055–1035	
	Forward	GCTCTTGGGTTGCATGAGTT	83	954–973	
	Reverse	GCTGTTTCACCCATCTGTTG		1036–1017	

bp—base pair; CDV—canine distemper virus; *—primers used for qualitative RT-PCR; ^#^ Puff et al., 2009 [34].

**Table 3:** CDV titers of different virus strains before and after neutralization by acidification (n = 3 per condition and group).

TCID_50_/mL		
	Range w/o Acidification	Range After Acidification
non-infected control	-	-
CDV-Ond pi	10^3^–10^4^	-
CDV-Ond^neon^ pi	10^3^–10^3.75^	-
CDV-Ond^neon-GM-CSF^ pi	10^3^–10^4^	-

CDV—canine distemper virus; GM-CSF—granulocyte and macrophage colony-stimulating factor; TCID_50_/mL—50% log10 tissue culture infectious dose per milliliter; w/o—without.

**Table 4:** Influence of acidification on the number of CDV nucleoprotein mRNA transcripts in culture supernatants obtained from DH82 cells with persistent CDV infections and non-infected controls (n = 4 per condition and group).

Infection Status	Number of CDV mRNA Transcripts in Supernatants without Acidification Median (Range)	Number of CDV mRNA Transcripts in Supernatants after Acidification Median (Range)
Non-infected	0 (0.00–0.00)	0 (0.00–0.00)
CDV-Ond pi	2841.86 (668.86–10154.25)	156.98 (93.92–495.60)
CDV-Ond^neon^ pi	4287.86 (1132.87–17252.94)	122.14 (103.97–237.24)
CDV-Ond^neon-GM-CSF^ pi	17508.73 (6657.71–87199.45)	821.93 (514.62–1481.72)

CDV-Ond—canine distemper virus strain Onderstepoort; pi—persistently infected; GM-CSF—granulocyte and macrophage colony-stimulating factor.

**Table 5:** Amount of CDV nucleoprotein within DH82 cell culture supernatants (n = 3 per condition and group).

Infection Status	CDV nucleoprotein IOD in Supernatants without Acidification Median (Range)	CDV nucleoprotein IOD in Supernatants after Acidification Median (Range)
Non-infected	0.00 (0.00–0.00)	0.00 (0.00–0.00)
CDV-Ond pi	11.05 (4.12–33.32)	13.24 (2.96–13.44)
CDV-Ond^neon^ pi	58.11 (45.88–112.42)	45.24 (42.21–124.92)
CDV-Ond^neon-GM-CSF^ pi	113.84 (84.48–155.00)	142.68 (99.00–154.71)

CDV-Ond—canine distemper virus strain Onderstepoort; pi—persistently infected; GM-CSF—granulocyte and macrophage colony-stimulating factor; IOD—integrated optical density

**Table 6:** Amount of GM-CSF within DH82 cell culture supernatants (n = 3 per condition and group).

Infection Status	GM-CSF IOD in Supernatants without Acidification Median (Range)	GM-CSF IOD in Supernatants after Acidification Median (Range)
Non-infected	0.00 (0.00–0.00)	0.00 (0.00–0.00)
CDV-Ond pi	0.00 (0.00–0.00)	0.00 (0.00–0.00)
CDV-Ond^neon^ pi	0.00 (0.00–0.00)	0.00 (0.00–0.00)
CDV-Ond^neon-GM-CSF^ pi	130.48 (62.98–150.29)	131.08 (45.29–153.36)

CDV-Ond—canine distemper virus strain Onderstepoort; pi—persistently infected; GM-CSF—granulocyte and macrophage colony-stimulating factor; IOD—integrated optical density

## Data Availability

All relevant data are included in the manuscript can be obtained from the authors upon reasonable request.
